# Energy-normalized ascorbic acid sono-dosimetry for assessing cavitation-derived oxidative activity

**DOI:** 10.1016/j.ultsonch.2026.108002

**Published:** 2026-08-10

**Authors:** Oualid Hamdaoui, Intissar Gasmi, Muthupandian Ashokkumar, Lahssen El Blidi, Abdulaziz Alghyamah

**Affiliations:** a Chemical Engineering Department, College of Engineering, King Saud University, 12372 Riyadh, Saudi Arabia; b Environmental Research Center (CRE), Annaba 23000, Algeria; c School of Chemistry, The University of Melbourne, Parkville, Victoria 3010, Australia

**Keywords:** Ascorbic acid, Sono-oxidation, Hydroxyl radical, Sonochemical dosimetry, Acoustic cavitation, Ultrasonic energy density

## Abstract

A UV–visible sonochemical dosimetry method based on ascorbic acid (AscA) sono-oxidation was developed to assess cavitation-derived oxidative activity in aerated aqueous solution. AscA was used as a fast-reacting probe for hydroxyl-radical-initiated oxidation and was stabilized against non-sonochemical oxidation by adding 0.2 M NaCl. Its consumption was monitored directly at 262 nm. Experiments were performed in a jacketed sonoreactor at 585, 860, and 1140 kHz using 300 mL of 100 µM AscA solution at 25 ± 1 °C. AscA depletion followed apparent zero-order kinetics under all investigated conditions, indicating that the measured rate was governed mainly by the quasi-steady generation and transfer of cavitation-derived oxidants rather than by the instantaneous AscA concentration. At 585 kHz, the apparent zero-order rate constant increased from 1.58 to 4.23 µM/min when the calorimetric intensity increased from 0.83 to 4.30 W/cm^2^. Energy-density normalization showed that the oxidative response decreased with increasing frequency, with G_AscA_ values of 2.23 × 10^−10^, 1.28 × 10^−10^, and 1.01 × 10^−10^ mol/J at 585, 860, and 1140 kHz, respectively. The sono-oxidation rate was pH-dependent, with an approximately invariant response between pH 4.8 and 8. Non-sonicated aerated controls showed no measurable AscA loss over the experimental time window, whereas under ultrasound the gas-atmosphere effect followed the order air > Ar ≫ N_2_. This distinction indicates that dissolved O_2_ enhanced oxygen-mediated sonochemical radical pathways rather than causing appreciable background oxidation of AscA. Comparison with H_2_O_2_ formation confirmed that AscA sono-dosimetry provides a complementary measure of the oxidative dose accessible to a dissolved radical scavenger. This method offers a simple, reproducible, and energy-normalized platform for comparing sonochemical activity in acoustic cavitation systems.

## 1 Introduction

Acoustic cavitation is the central phenomenon responsible for the chemical effects of ultrasound in liquids. When ultrasonic waves propagate through an aqueous medium, pre-existing gas nuclei can grow, oscillate, and collapse violently, producing localized hot spots characterized by transiently high temperature and pressure [1], [2], [3]. These extreme microenvironments promote the thermal dissociation of water vapor and dissolved gases, generating not only hydroxyl radicals (HO^•^) and hydrogen atoms (H^•^), but also atomic oxygen, H_2_O_2_, and other oxygen-derived species [1], [2], [3], [4]. Atomic oxygen may originate from the high-temperature dissociation of water-derived species and, in oxygen-containing bubbles, from the dissociation of O_2_. Numerical simulations indicate that appreciable amounts of O atoms can be formed during violent bubble collapse and that O atoms may become a major intra-bubble oxidant in sufficiently hot gaseous bubbles. Nevertheless, the electronic state, concentration, and lifetime of O atoms transferred into the surrounding liquid remain poorly established [5]. In aerated water, the primary species leaving the cavitation zone may also react with dissolved O_2_ to form secondary oxygen-derived oxidants, while HO^•^ recombination produces H_2_O_2_.

Although aqueous sonochemical reactions are frequently interpreted in terms of oxidative species, collapsing cavitation bubbles simultaneously generate reducing species. In particular, H^•^ atoms formed through the high-temperature dissociation of water vapor possess strong reducing character and may escape into the bubble–solution interfacial region or surrounding liquid. Their survival depends on bubble composition because H^•^ can be consumed inside oxygen-containing bubbles through reactions with O_2_ and HO^•^, producing HO_2_^•^, H_2_, and other species. Consequently, an aqueous solute may experience competing oxidative and reductive reaction pathways during ultrasonication [5].

Sonochemical activity is not controlled solely by the nominal electrical power supplied to a transducer. It also depends on the effective calorimetric power dissipated in the liquid, ultrasonic frequency, reactor geometry, liquid volume, temperature, dissolved gas composition, and solution chemistry [4], [6], [7]. For this reason, chemical dosimetry has become an important tool for evaluating cavitation-derived radical production. Several methods have been used, including iodide/Weissler, Fricke, terephthalic acid, salicylic acid, 4-nitrophenol, Ti(IV), peroxynitrite formation and hydrogen peroxide accumulation methods [6], [7], [8], [9], [10], [11], [12]. Each method, however, probes a different part of the radical reaction network. For example, terephthalate and salicylic acid methods detect hydroxylated products, whereas hydrogen peroxide accumulation represents only the fraction of hydroxyl radicals that recombine into a stable molecular product [8], [9], [10]. Consequently, different dosimeters can produce different apparent radical yields under the same ultrasonic conditions.

Ascorbic acid (AscA) is an attractive candidate for sonochemical dosimetry because of its high aqueous solubility, well-established redox chemistry, strong UV absorbance, and rapid reaction with hydroxyl radicals. AscA/ascorbate undergoes one-electron oxidation to form the resonance-stabilized ascorbate radical, followed by further oxidation to dehydroascorbic acid and downstream degradation products [13], [14], [15]. Reported rate constants for the reaction of hydroxyl radicals with AscA/ascorbate are close to the diffusion-controlled limit, on the order of 10^10^ M^−1^ s^−1^ [13], [16]. Therefore, AscA can efficiently scavenge short-lived oxidants escaping from cavitation zones.

A practical challenge, however, is that aqueous AscA is unstable under oxygen, light, heat, alkaline pH, and trace-metal conditions [15], [17]. In pure aerated water, the characteristic UV band of AscA near 262 nm can decrease with standing time even in the absence of ultrasound. Earlier dosimetry work showed that several additives can stabilize AscA solutions, among which NaCl is particularly effective [17]. In the present work, 0.2 M NaCl was used as an operational stabilizer against non-sonochemical AscA loss during the experimental time window. Previous studies have shown that NaCl can stabilize dilute aqueous AscA solutions and, under certain conditions, inhibit trace-metal-catalyzed aerobic oxidation [17], [18], [19], [20], [21]. This protection does not result from direct scavenging of O_2_ by NaCl but may involve chloride-induced modification of catalytic metal speciation together with a moderate reduction in dissolved-O_2_ availability through salting out.

The novelty of this study lies in implementing NaCl-stabilized AscA sono-oxidation as a UV–visible, apparent zero-order, energy-normalized dosimetry method for acoustic cavitation. The method was systematically evaluated at 585, 860, and 1140 kHz under different ultrasonic intensities, initial pH values, and gas atmospheres. The response was also compared with hydrogen peroxide production to distinguish direct oxidative-dose probing from radical recombination products. The objectives were to: (i) demonstrate UV spectral evidence for AscA consumption during ultrasonication; (ii) establish apparent zero-order kinetic behavior; (iii) evaluate intensity, frequency, and energy–density effects; (iv) determine suitable pH and gas-atmosphere conditions; and (v) provide a mechanistic and analytical framework for using AscA as a sonochemical dosimeter.

The main contribution of this work is the development of a NaCl-stabilized AscA sono-dosimetry approach that converts the zero-order depletion of a dissolved radical scavenger into both time-normalized and energy-normalized indices of cavitation-derived oxidative activity. Unlike dosimeters based only on accumulated products such as H_2_O_2_, the proposed method directly probes the oxidative dose accessible to a solute in the aqueous phase. This makes the method useful for comparing sonochemical reactors, frequencies, intensities, pH windows, and gas atmospheres under a common calorimetric-energy basis.

## 2 Materials and methods

### 2.1 Chemicals

Ascorbic acid, sodium chloride, hydrochloric acid, sodium hydroxide, potassium iodide, and ammonium heptamolybdate tetrahydrate were analytical-grade reagents purchased from Sigma-Aldrich and used as received. All solutions were prepared using ultrapure water. Fresh AscA solutions were prepared immediately before use. Sodium chloride was added at 0.2 M as an operational stabilizer against non-sonochemical AscA loss during standing and ultrasonication experiments, following the concentration previously proposed for UV-based AscA dosimetry [17]. Its effectiveness was verified experimentally using non-sonicated aerated controls, as described in Section 2.6. The main physicochemical properties of AscA relevant to the present dosimetry method are summarized in Table 1.

**Table 1:** Main physicochemical properties of AscA relevant to the present dosimetry method.

Molecule	Ascorbic acid
CAS Number	50-81-7
Chemical structure	
Molecular formula	C_6_H_8_O_6_
Synonyms	Ascorbic acid, L-ascorbic acid, vitamin C
Solubility	0.33 g/mL
Vapor pressure (mmHg)	9.28 × 10^−11^ at 25 °C
Octanol-water partition coefficient, log K_ow_	−1.85
pK_a_	pK_a1_ = 4.1; pK_a2_ = 11.8
Maximum absorption wavelength (λ_max_)	262 nm

### 2.2 Sonoreactor and operating conditions

Sonochemical experiments were carried out in a jacketed cylindrical glass sonoreactor with a total capacity of 500 mL. Ultrasonic irradiation was generated using a high-frequency Meinhardt Ultrasonics E/805/T/M piezoelectric transducer mounted at the bottom of the reactor. The transducer had an active diameter of 5.3 cm, corresponding to an active area of 22.1 cm^2^. Three discrete frequencies were used: 585, 860, and 1140 kHz.

For all experiments, the working liquid volume was fixed at 300 mL. The solution temperature was maintained at 25 ± 1 °C by circulating cooling liquid through the reactor jacket. Unless otherwise stated, experiments were conducted under naturally aerated conditions. The applied calorimetric intensities were 0.83, 2.64, and 4.30 W/cm^2^ at 585 kHz; 1.04, 2.83, and 5.19 W/cm^2^ at 860 kHz; and 1.23, 3.74, and 5.33 W/cm^2^ at 1140 kHz.

### 2.3 Calorimetric determination of ultrasonic power

The effective ultrasonic power delivered to the liquid was determined calorimetrically. A type-K thermocouple was placed at the mid-height of the liquid volume, and the temperature increase was recorded during the first 300 s of irradiation. Before calorimetric measurements, the cooling liquid was drained from the reactor jacket to minimize external heat losses.

The calorimetric power, P_cal_, was calculated from the initial slope of the temperature–time profile according to:

(1) $$P_{cal}=mC_{p}{\left(\frac{dT}{dt}\right)}_{t\to 0}$$

where m is the mass of the irradiated liquid, C_p_ is the specific heat capacity of water, and ${\left(\frac{dT}{dt}\right)}_{t\to 0}$ is the initial rate of temperature increase. This approach assumes that acoustic energy dissipated in the liquid is converted into heat during the short calorimetric measurement [4], [15], [22].

The ultrasonic intensity, I, was obtained by normalizing the calorimetric power to the active transducer area:

(2) $$I=\frac{P_{cal}}{A}$$

where A is the active surface area of the transducer.

The volumetric power density, P_V_, was calculated as:

(3) $$P_{V}=\frac{P_{cal}}{V}$$

where V is the irradiated liquid volume.

The ultrasonic energy density, E_V_, was calculated as:

(4) $$E_{V}=\frac{P_{cal}t}{V}$$

where t is the ultrasonication time.

### 2.4 Hydrogen peroxide production

Hydrogen peroxide formation during ultrasonication was determined by iodometry [6]. During ultrasonic irradiation, 200 µL aliquots were periodically withdrawn and mixed in a quartz cuvette with 1.0 mL of 0.1 M KI and 20 µL of 0.01 M ammonium heptamolybdate. After 5 min of reaction, absorbance was measured at 352 nm using a Biochrom WPA Lightwave II UV–visible spectrophotometer. The rate of H_2_O_2_ formation was used as a complementary indicator of hydroxyl radical recombination. Importantly, KI and ammonium heptamolybdate were added only after the aliquot had been withdrawn from the irradiated solution; neither reagent was present in the sonoreactor during ultrasonication. Consequently, the iodometric response reflects accumulated H_2_O_2_ and cannot include direct oxidation of I^−^ by transient cavitation-derived HO^•^ or O atoms.

### 2.5 AscA sono-oxidation experiments

AscA sono-oxidation was used as an indirect dosimetric probe of cavitation-derived oxidizing species. Solutions were prepared by dissolving AscA in ultrapure water containing 0.2 M NaCl to obtain an initial AscA concentration of 100 µM. The NaCl-stabilized AscA solution was introduced into the sonoreactor and equilibrated to 25 ± 1 °C before irradiation.

At selected ultrasonication times, approximately 1 mL of solution was withdrawn and transferred to a quartz cuvette. The residual AscA concentration was determined by measuring absorbance at 262 nm. After each measurement, the analyzed sample was returned to the reactor to maintain constant liquid volume and preserve the applied ultrasonic power density. For UV spectral monitoring, 78 µM AscA solutions were sonicated under selected conditions and spectra were recorded between 200 and 300 nm.

Only the absorbance at 262 nm was used to calculate residual AscA concentrations and kinetic parameters. The short-wavelength region below approximately 230 nm was examined only qualitatively because multiple sonochemical products may contribute to the UV absorbance in this region. No attempt was therefore made to identify or quantify individual products from these spectra.

### 2.6 Effect of initial pH

The effect of initial pH was investigated at 585 kHz and 4.30 W/cm^2^ using 300 mL of 100 µM AscA solution at 25 ± 1 °C. The natural pH of the AscA solution containing 0.2 M NaCl was approximately 4.8. For pH-dependent experiments, the initial pH was adjusted using HCl or NaOH before ultrasonication. Non-sonicated controls were conducted under naturally aerated conditions at 25 ± 1 °C and analyzed at the same sampling times as the sonicated solutions. These controls were used to evaluate non-sonochemical AscA oxidation, including any background reaction with dissolved molecular O_2_. No measurable decrease in absorbance at 262 nm was observed over the experimental time window; consequently, background aerobic oxidation was considered negligible relative to the sono-oxidation signal.

### 2.7 Effect of dissolved gas

The effect of gas atmosphere was examined at 585 kHz and 4.30 W/cm^2^, natural pH, and 25 ± 1 °C. Experiments were performed under air, argon, and nitrogen. For Ar and N_2_ experiments, the solution was purged with the selected gas at 100 mL/min for 20 min before ultrasonication, and gas bubbling was continued during ultrasonication. Air experiments were performed under naturally aerated conditions.

The solution pH was measured before and after ultrasonication under each gas atmosphere. Under air, only a negligible pH variation was observed over the experimental irradiation period. The natural pH of approximately 4.8 was governed primarily by the acid–base equilibrium of AscA in the 0.2 M NaCl medium.

A control experiment was also performed under air bubbling at the same flow rate used for Ar and N_2_ purging. The AscA sono-oxidation profile obtained under air bubbling was indistinguishable, within experimental uncertainty, from that obtained under naturally aerated conditions. Therefore, the air condition reported in the main gas-comparison experiment represents the aerated system reliably.

### 2.8 Kinetic analysis and data treatment

AscA sono-oxidation was evaluated from the normalized concentration ratio C/C_0_, where C_0_ and C are the initial and time-dependent AscA concentrations, respectively. Concentration was determined from absorbance at 262 nm, assuming Beer-Lambert proportionality within the investigated range. A calibration curve of AscA at 262 nm was linear over 0–100 µM with R^2^ = 0.9999.

The kinetic profiles were analyzed using an apparent zero-order model because C/C_0_ decreased linearly with ultrasonication time. The resulting k_0,app_ represents the net apparent disappearance rate of UV-detectable AscA. It includes oxidative consumption of AscA minus any regeneration of AscA from oxidized intermediates through cavitation-derived reducing species. Because these opposing contributions were not measured independently, k_0,app_ should not be interpreted as an elementary or species-specific oxidation rate constant.

(5) $$-\frac{dC_{AscA}}{dt}=k_{0,app}$$

where k_0,app_ is the apparent zero-order net AscA-disappearance rate constant.

Integration gives:

(6) $$C=C_{0}-k_{0,app}t$$

or, in normalized form:

(7) $$\frac{C}{C_{0}}={1-(k}_{0,app}/C_{0})t$$

Thus, k_0,app_ was obtained from the slope of the linear C versus t plot. For normalized plots, the slope of C/C_0_ versus time corresponds to −k_0,app_/C_0_.

The energy-normalized dosimetric response was calculated as:

(8) $$\eta_{AscA,E}=-\frac{d(C/C_{0})}{dE_{V}}$$

or equivalently:

(9) $$\eta_{AscA,E}=\frac{k_{0,app}}{C_{0}\left({0.06P}_{V}\right)}$$

where P_V_ is expressed in W/L, C_0_ = 100 µM, and the factor 0.06 converts P_V_ from W/L to kJ/L·min (1 W/L = 0.06 kJ/L·min).

All experiments were performed in triplicate, and the relative standard deviation (RSD) did not exceed 2.5%.

## 3 Results and discussion

### 3.1 Stabilization and solution behavior of AscA in the presence of NaCl

Aqueous AscA is susceptible to degradation in the presence of dissolved oxygen, light, heat, alkaline pH, and trace impurities [15], [17], [19], [20]. Therefore, non-sonochemical loss must be minimized before AscA can be used as a sonochemical dosimeter. In pure aerated water, the AscA absorption band at 262 nm decreased during standing, whereas addition of 0.2 M NaCl stabilized the 100 µM AscA solution over the experimental time window. A similar stabilizing effect of 0.2 M NaCl was previously reported for UV-based AscA dosimetry [17].

The protection afforded by NaCl should not be interpreted as direct chemical scavenging or deactivation of molecular O_2_. Aerobic AscA oxidation is relatively slow in carefully purified aqueous media but can be strongly accelerated by trace Cu and Fe species through metal–ascorbate redox cycling. Earlier studies reported inhibition of Cu-catalyzed AscA oxidation by NaCl under certain conditions, while subsequent kinetic studies showed that Cl^−^ modifies the speciation and reactivity of the Cu(I)/Cu(II)–ascorbate–O_2_ system [18], [19], [20], [21]. Chloride coordination can alter the concentrations and redox properties of the metal species that catalyze electron transfer from ascorbate to O_2_, thereby changing the background oxidation rate.

A second contribution is the salting-out of molecular oxygen. Dissolved NaCl decreases the equilibrium solubility and activity of O_2_ in water according to Setschenow-type behavior [18]. At 0.2 M NaCl, this effect is expected to be moderate and is unlikely to account alone for the observed stabilization, but it decreases the O_2_ available to sustain background aerobic oxidation. Thus, the protection observed here is plausibly attributable to the combined modification of trace-metal-mediated oxidation pathways and a moderate reduction in effective dissolved-O_2_ availability.

The chloride effect on metal-catalyzed AscA oxidation is known to depend on pH, chloride concentration, metal concentration, and metal–ligand speciation; it is therefore not universally inhibitory. Because dissolved O_2_, trace-metal concentrations, and metal speciation were not measured in the present study, the relative contributions of these mechanisms cannot be quantified. Nevertheless, the non-sonicated aerated control showed no measurable decrease in the AscA band at 262 nm over the experimental time window, empirically confirming that background aerobic oxidation was negligible in the standardized 0.2 M NaCl medium. Dissolved O_2_ nevertheless remains mechanistically relevant during ultrasonication because it converts primary cavitation-derived species into HO_2_^•^/O_2_^•−^ and thereby extends the sonochemical radical network.

The acid-base distribution of AscA is also relevant. AscA has pK_a_ values of approximately 4.1 and 11.8 (Table 1). At the natural pH of the NaCl-stabilized solution, about 4.8, AscA exists mainly as the monoanionic ascorbate form. The ascorbate fraction can be estimated from:

(10) $$f_{{AscH}^{-}}=\frac{10^{pH-pK_{a}}}{1+10^{pH-pK_{a}}}$$

Using pH 4.8 and pK_a_ 4.1, the solution contains approximately 83.4% ascorbate anion and 16.6% protonated AscA. Because AscA has low vapor pressure and strong water affinity (Table 1), oxidation is expected to occur mainly in the interfacial and bulk aqueous regions by reaction with cavitation-derived oxidants, rather than by volatilization into the cavitation bubble.

Accordingly, all sonochemical comparisons in this study were performed at fixed NaCl concentration, so that changes in AscA depletion reflect changes in ultrasonic operating conditions rather than changes in solution stabilization.

It should be emphasized that 0.2 M NaCl defines the standardized medium of the present dosimetry method. Although NaCl was introduced to stabilize AscA against non-sonochemical oxidation, the fixed ionic strength may also influence bulk physicochemical properties relevant to acoustic cavitation. Therefore, the reported k_0,app_, $\eta_{AscA,E}$, and G_AscA_ values should be interpreted as dosimetric responses in the NaCl-stabilized AscA medium. This does not affect comparisons within the present study because NaCl concentration was kept constant in all AscA experiments.

### 3.2 UV spectral evidence for AscA sono-oxidation

**Fig. 1:**
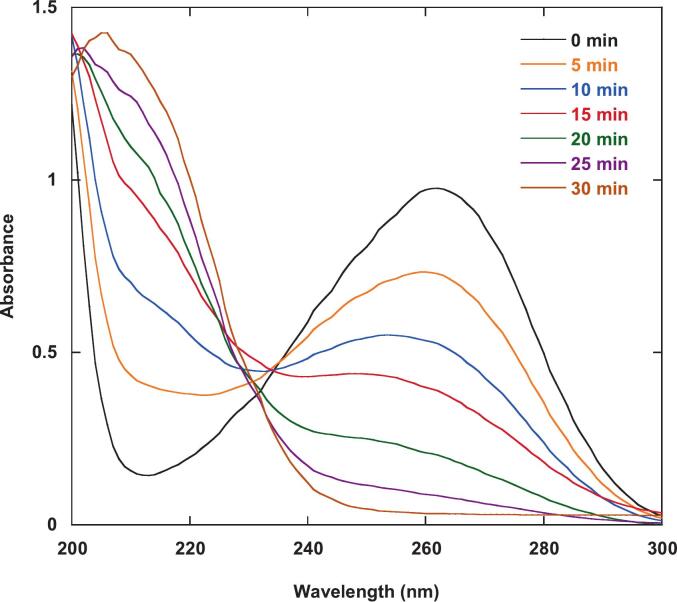
Changes in UV absorption spectra observed during sono-oxidation of AscA (conditions: AscA initial concentration: 78 µM, frequency: 860 kHz, ultrasonication intensity: 5.19 W/cm^2^, volume: 300 mL, temperature: 25 °C, pH: 4.8 (natural)).

Fig. 1 shows the UV spectral evolution during ultrasonication of 78 µM AscA at 860 kHz and 5.19 W/cm^2^. The characteristic AscA absorption band near 262 nm progressively decreased with ultrasonication time. This wavelength is appropriate for monitoring AscA under weakly acidic conditions because AscA shows strong UV absorbance around 262 nm near pH 4–5.

The progressive decrease in absorbance at 262 nm indicates consumption of the parent AscA chromophore during ultrasonication. Oxidation of the electron-rich enediol structure produces the ascorbyl radical, dehydroascorbic acid, and subsequent degradation products [13], [14], [15]. However, the increase in absorbance observed in the short-wavelength region cannot be assigned to specific AscA oxidation products on the basis of the present UV spectra alone. High-frequency sonolysis of aerated water is known to generate several dissolved products, including nitrogen-containing species under appropriate conditions [23], and some inorganic oxyanions as well as AscA oxidation products absorb in the lower-UV region [24]. Nevertheless, neither NO_2_^−^, NO_3_^−^ nor individual AscA oxidation products were selectively quantified in the present experiments. Accordingly, no compositional assignment or quantitative interpretation of the absorbance below approximately 230 nm is made here. Selective analytical methods, such as ion chromatography for inorganic nitrogen oxyanions and chromatographic or mass-spectrometric analysis for organic oxidation products, would be required to resolve the species contributing to this region. The lower-UV spectral evolution is therefore considered only as qualitative evidence that additional UV-absorbing species are formed during ultrasonication and is not used in the AscA dosimetric analysis.

In contrast, the characteristic AscA band at 262 nm decreased continuously with irradiation time and was used as the analytical signal for determining residual AscA concentration. The present dosimetric conclusions are therefore based on depletion of the parent AscA signal rather than on identification of the products responsible for the lower-wavelength absorbance.

### 3.3 Effect of ultrasonic intensity on AscA sono-oxidation

**Fig. 2:**
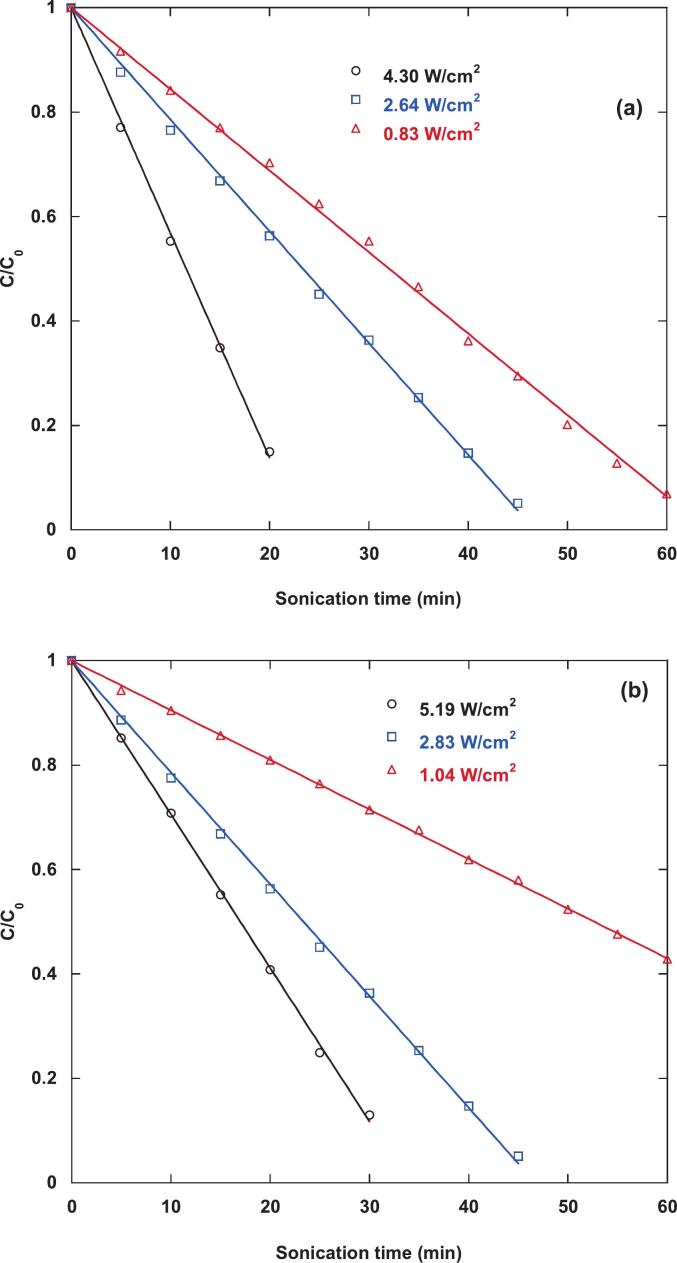

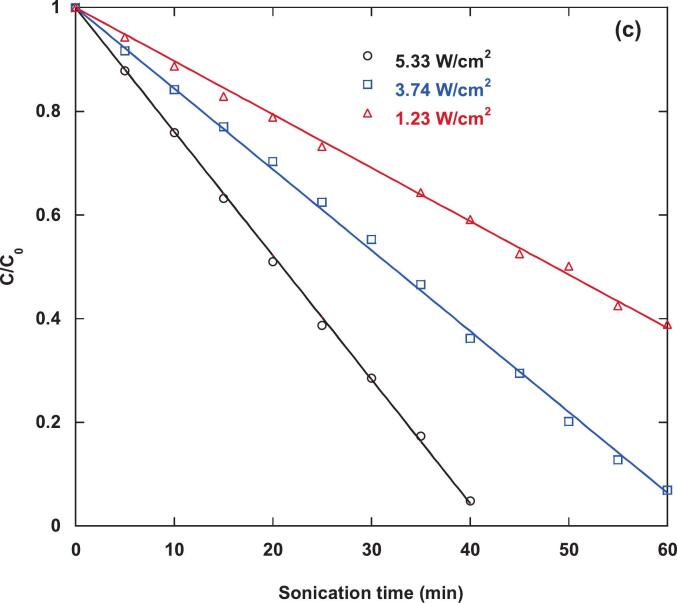
Sono-oxidation of AscA at 585 kHz (a), 860 kHz (b), and 1140 kHz (c) in water at various ultrasonication intensities (conditions: AscA initial concentration: 100 µM, 585 kHz (0.83, 2.64, and 4.3 W/cm^2^), 860 kHz (1.04, 2.83, and 5.19 W/cm^2^), 1140 kHz (1.23, 3.74, and 5.33 W/cm^2^), volume: 300 mL, temperature: 25 °C, pH: 4.8 (natural)).

The effect of ultrasonic intensity was evaluated at 585, 860, and 1140 kHz. As shown in Fig. 2a–c, AscA concentration decreased linearly with ultrasonication time under all investigated conditions, confirming apparent zero-order behavior. Increasing ultrasonic intensity accelerated AscA depletion at each frequency.

At 585 kHz, k_0,app_ increased from 1.58 to 2.09 and 4.23 µM/min when the intensity increased from 0.83 to 2.64 and 4.30 W/cm^2^, respectively. At 860 kHz, the rate increased from 0.94 to 2.10 and 2.94 µM/min when intensity increased from 1.04 to 2.83 and 5.19 W/cm^2^. At 1140 kHz, the rate increased from 1.01 to 1.58 and 2.37 µM/min as the intensity increased from 1.23 to 3.74 and 5.33 W/cm^2^.

The increase in AscA oxidation rate with intensity reflects enhanced cavitation activity. Higher intensity increases the acoustic energy dissipated into the liquid, which can increase both the number of active cavitation bubbles and the collapse intensity of individual bubbles. These effects increase water sonolysis and oxidant generation. The higher rate at higher intensity therefore reflects an increase in the effective oxidative flux accessible to the dissolved probe.

The measured k_0,app_ nevertheless represents a net response. Ultrasound generates reducing H^•^ atoms concurrently with oxidizing species, and H^•^ may partly regenerate AscA from its oxidized radical intermediates. Therefore, an increase in k_0,app_ indicates that oxidative AscA consumption increased relative to any concurrent reductive regeneration; it does not quantify the gross oxidant-production rate independently of the reducing branch.

Table 2 summarizes the apparent zero-order kinetic parameters, calorimetric power, volumetric power density, regression coefficients, and energy-normalized dosimetric responses.

**Table 2:** Apparent zero-order kinetic and energy-normalized dosimetric parameters for AscA sono-oxidation under different ultrasonic and gas-atmosphere conditions.

Parameter	Frequency (kHz)	Intensity (W/cm^2^)	pH	Gas	P_cal_ (W)	P_V_ (W/L)	R^2^	k_0,app_ (µM/min)	$\eta_{AscA,E}$(10^−3^ L/kJ)
Frequency/intensity	585	0.83	4.8	Air	18.34	61.1	0.9985	1.58	4.31
	585	2.64	4.8	Air	58.34	194.5	0.9993	2.09	1.79
	585	4.30	4.8	Air	95.03	316.8	0.9990	4.23	2.23
	860	1.04	4.8	Air	22.98	76.6	0.9994	0.94	2.05
	860	2.83	4.8	Air	62.54	208.5	0.9994	2.10	1.68
	860	5.19	4.8	Air	114.70	382.3	0.9992	2.94	1.28
	1140	1.23	4.8	Air	27.18	90.6	0.9982	1.01	1.87
	1140	3.74	4.8	Air	82.65	275.5	0.9986	1.58	0.96
	1140	5.33	4.8	Air	117.79	392.6	0.9993	2.37	1.01
Dissolved gas	585	4.30	4.8	Air	95.03	316.8	0.9990	4.23	2.23
	585	4.30	4.8	Ar	95.03	316.8	0.9996	2.72	1.43
	585	4.30	4.8	N_2_	95.03	316.8	0.9899	0.88	0.46

P_cal_ = I × A, where A = 22.1 cm^2^, and P_V_ = P_cal_/V, where V = 300 mL. The apparent zero-order rate constant was calculated from the slope of the linear concentration–time profile using C_0_ = 100 µM.

### 3.4 Frequency dependence and energy-normalized dosimetry

**Fig. 3:**
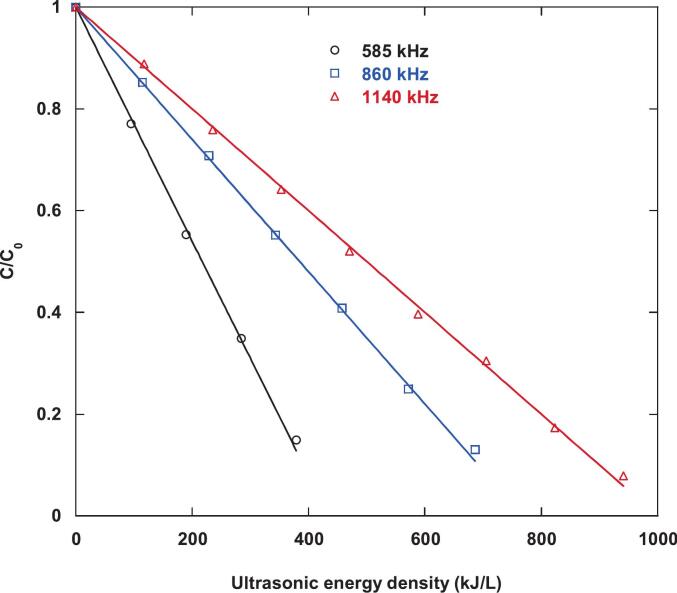
Sono-oxidation of AscA as a function of the ultrasonic energy density for different frequencies using the highest investigated calorimetric intensity at each frequency: 585 kHz, 4.30 W/cm^2^; 860 kHz, 5.19 W/cm^2^; and 1140 kHz, 5.33 W/cm^2^. (conditions: AscA initial concentration: 100 µM, volume: 300 mL, temperature: 25 °C, pH: 4.8 (natural)).

The effect of ultrasonic frequency was assessed by comparing the AscA decay at 585, 860, and 1140 kHz and by normalizing the response with respect to ultrasonic energy density. Fig. 3 shows C/C_0_ as a function of E_V_ at the highest intensity tested for each frequency. At the highest tested intensity for each frequency, the energy-normalized AscA response followed the order 585 > 860 > 1140 kHz. The same ordering is evident from the corresponding energy-normalized responses in Table 2. Because the calorimetric intensities were not identical across frequencies, this trend should be interpreted within the investigated reactor configuration and operating ranges rather than as an intrinsic frequency effect at strictly matched acoustic intensity. The results nevertheless demonstrate that the total acoustic energy supplied to the liquid is not sufficient by itself to define the oxidative dose. This agrees with previous work showing that sonochemical reaction efficiency depends jointly on ultrasonic frequency, power, and liquid volume rather than on acoustic energy input alone [25]. Instead, the efficiency with which acoustic energy is converted into chemically active cavitation depends strongly on frequency.

At lower frequency, bubbles have more time to expand during the rarefaction phase before collapse. Larger expansion ratios can produce more energetic collapses and higher single-bubble radical yields [2], [7], [26]. At higher frequencies, bubbles are generally smaller and oscillate over shorter periods. Although the number of cavitation events may increase, the radical yield per bubble and radical escape efficiency may decrease [7], [26]. The present data suggest that, within the investigated frequency range and reactor geometry, the net chemical productivity of cavitation decreases as frequency increases. This trend is consistent with numerical single-bubble calculations showing decreasing oxidant production with increasing driving frequency and with experimental studies demonstrating that frequency-dependent reactor-scale sonochemical yields reflect the combined effects of single-bubble dynamics and the population of active bubbles [26], [27]. Changes in radical transport from the bubble–solution interface to the bulk liquid may also contribute, but this effect was not measured directly in the present study.

The energy-specific yield for AscA sono-oxidation is given by:

(11) $$G_{AscA}=\frac{k_{0.app}V}{P_{cal}\times 60}$$

where k_0,app_ is expressed in mol/L·min, V is in L, and P_cal_ is in J/s.

The corresponding energy-specific yields for AscA sono-oxidation were summarized in Table 3. These values confirm that, at the highest investigated intensity for each frequency, the energy-specific AscA oxidation yield followed the order 585 > 860 > 1140 kHz under the present reactor configuration.

**Table 3:** Energy-specific yields for AscA sono-oxidation.

Frequency (kHz)	Highest intensity used (W/cm^2^)	G_AscA_ (mol/J)
585	4.30	2.23 × 10^−10^
860	5.19	1.28 × 10^−10^
1140	5.33	1.01 × 10^−10^

### 3.5 Comparison with hydrogen peroxide formation

Hydrogen peroxide formation was measured in an AscA-free 0.2 M NaCl solution under the reference condition of 585 kHz and 4.30 W/cm^2^, allowing matrix-matched comparison between AscA depletion and recombination-product dosimetry. AscA was omitted from the H_2_O_2_ experiment because it can consume cavitation-derived oxidants and may also react with H_2_O_2_, which would interfere with direct assessment of radical recombination products. The iodometric procedure employed here should be distinguished from conventional KI sonochemical dosimetry. When I^−^ is present during ultrasonic irradiation, it can be oxidized by HO^•^ and possibly by atomic oxygen transferred from the cavitation bubbles. The latter pathway has been represented by the overall reaction O + 2I^−^ + 2H^+^ → I_2_ + H_2_O, and has been proposed to explain cases in which sonochemical I_2_ production exceeds that expected from H_2_O_2_ accumulation alone [4], [5]. In the present experiments, however, KI and ammonium heptamolybdate were introduced only after withdrawal of the irradiated aliquot. Because cavitation-derived O atoms and HO^•^ are transient species, they were no longer available to react directly with I^−^ during the analytical step. Thus, the measured iodometric signal represents stable H_2_O_2_ accumulated in the AscA-free solution rather than the combined oxidation of I^−^ by H_2_O_2_, HO^•^, and O atoms. Under these conditions, H_2_O_2_ accumulated at 6.99 µM/min, corresponding to $G_{H_{2}O_{2}}$ = 3.69 × 10^−10^ mol/J. This value was higher than the AscA oxidation yield, G_AscA_ = 2.23 × 10^−10^ mol/J, obtained under the same acoustic condition. Because H_2_O_2_ accumulation and AscA depletion represent chemically distinct endpoints with different reaction stoichiometries, their numerical G values should not be interpreted as a direct radical mass balance; rather, the two measurements provide complementary operational measures of sonochemical activity. This difference is expected because the post-irradiation iodometric measurement quantifies stable H_2_O_2_ accumulated from radical recombination, whereas AscA depletion reflects the fraction of cavitation-derived oxidants that reaches and reacts with a dissolved scavenger. The latter fraction is expected to be dominated by HO^•^-initiated oxidation but may also include contributions from O atoms and secondary oxygen-derived species.

### 3.6 Influence of initial pH

**Fig. 4:**
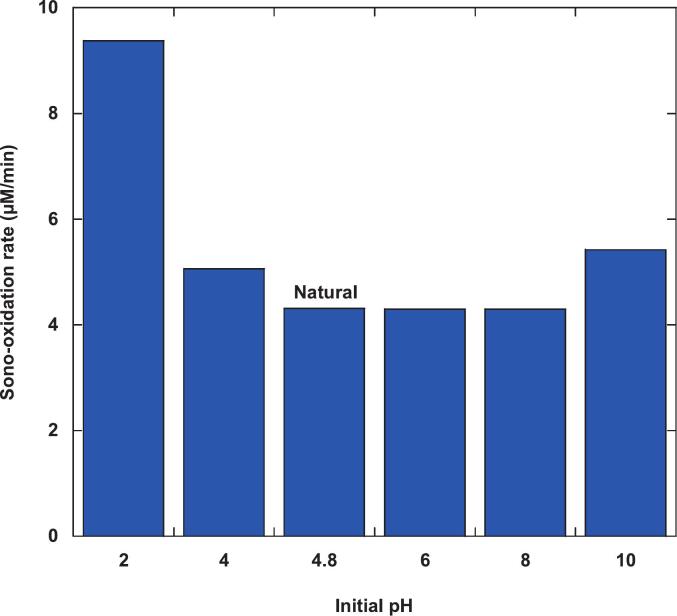
Sono-oxidation rate of AscA as a function of initial pH (conditions: AscA initial concentration: 100 µM, frequency: 585 kHz, intensity: 4.3 W/cm^2^, volume: 300 mL, temperature: 25 °C).

The influence of initial pH was investigated at 585 kHz and 4.30 W/cm^2^. As shown in Fig. 4, the AscA sono-oxidation rate was highest at pH 2, decreased sharply at pH 4, remained nearly constant between pH 4.8 and 8, and increased moderately at pH 10.

The UV–visible absorption spectra of the initial AscA solutions at pH 2, 4, 4.8, 6, 8, and 10 were essentially superimposable, with no measurable displacement of the characteristic absorption band or appreciable variation in absorbance at 262 nm. Therefore, the observed pH-dependent differences in the sono-oxidation rate cannot be attributed to changes in the analytical absorption response of AscA.

At pH 2, AscA is present mainly in its protonated form. This form is less hydrophilic than the ascorbate anion and may have a greater tendency to approach the cavitation bubble–solution interface, where the local concentration of primary radicals is high. Therefore, the enhanced oxidation rate at pH 2 is attributed mainly to changes in AscA speciation and interfacial accessibility to cavitation-derived oxidants. Because no direct radical-specific measurement was performed at each pH, this interpretation should be considered operational rather than species-specific.

At pH 4, the fraction of ascorbate anion increases, decreasing the tendency of the substrate to accumulate at the bubble interface. Between pH 4.8 and 8, AscA is present mainly as the monoanionic ascorbate form and remains predominantly in the bulk aqueous phase. In this pH range, oxidation occurs mainly through reaction with hydroxyl radicals diffusing from the cavitation zone into the interfacial and bulk liquid regions. The nearly constant rates between pH 4.8 and 8 indicate that this range is suitable for reproducible AscA dosimetry.

At pH 10, the moderate increase in rate is more conservatively attributed to the higher chemical lability of ascorbate and its oxidation products under alkaline conditions, together with possible changes in oxygen-derived radical chemistry during aerated sonolysis [11]. Since secondary oxidants were not directly quantified as a function of pH, the pH 10 response should be interpreted as an operational increase in the AscA dosimetric signal rather than as evidence for a specific additional oxidizing species.

The pH of the present AscA solution remained essentially unchanged during the experimental irradiation period. Because the AscA-depletion rate was nearly constant between pH 4.8 and 8, the small pH variation observed under air was not expected to affect the dosimetric response appreciably.

Therefore, for comparative dosimetry, AscA sono-oxidation should preferably be performed in the pH range 4.8–8. Conveniently, the natural pH of 100 µM AscA prepared in 0.2 M NaCl falls within this range.

### 3.7 Effect of dissolved gas atmosphere

**Fig. 5:**
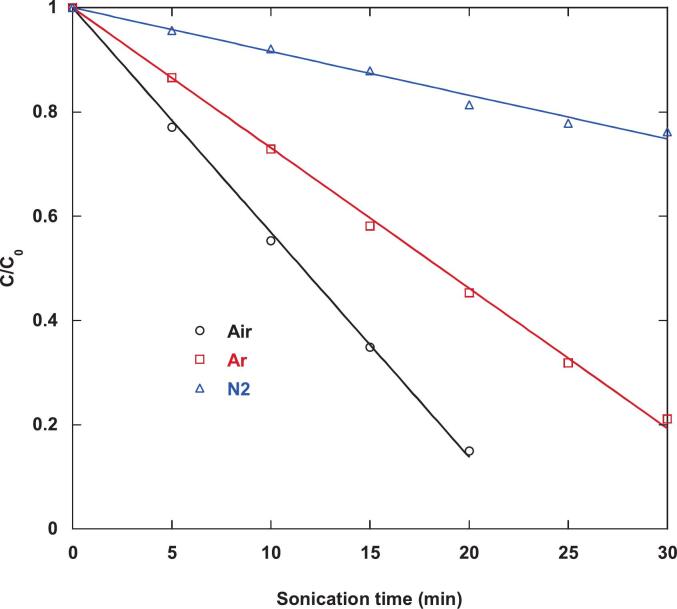
Impact of gases on the kinetics of AscA sono-oxidation (conditions: AscA initial concentration: 100 µM, frequency: 585 kHz, intensity: 4.3 W/cm^2^, volume: 300 mL, temperature: 25 °C, pH: 4.8 (natural)).

Dissolved gas atmosphere strongly affected AscA sono-oxidation. As shown in Fig. 5, the oxidation rate followed the order: Air > Ar ≫ N_2_. At 585 kHz and 4.30 W/cm^2^, the apparent zero-order rate constants were 4.23, 2.72, and 0.88 µM/min under air, Ar, and N_2_, respectively.

The possible influence of gas-dependent pH evolution was also examined. The pH of the aerated AscA solution remained close to its initial natural value of approximately 4.8 throughout ultrasonication, with only a negligible variation. Because the AscA dosimetric response was nearly constant between pH 4.8 and 8, this small pH change could not account for the substantially higher depletion rate under air. The observed order air > Ar ≫ N_2_ therefore reflects predominantly gas-dependent cavitation and O_2_-mediated radical chemistry rather than a secondary pH effect.

It is important to note that bubbling air through the solution at the same flow rate used for Ar and N_2_ produced the same AscA oxidation profile as the naturally aerated solution, within experimental uncertainty. Thus, the higher rate observed under air cannot be attributed to the absence of bubbling in the aerated experiment, but rather to the oxygen-containing atmosphere and the associated oxygen-mediated radical pathways.

Importantly, the higher AscA oxidation rate under air should not be interpreted as evidence that dissolved ground-state O_2_ directly oxidized AscA at a rate comparable to the sonochemical reaction. The absence of measurable AscA loss in the non-sonicated aerated control demonstrates that background aerobic oxidation was negligible over the experimental time window. Under ultrasonic irradiation, molecular O_2_ instead participates in the cavitation-induced radical network. In particular, O_2_ rapidly intercepts cavitation-derived H^•^ to form HO_2_^•^ according to Eq. (12), while the HO_2_^•^/O_2_^•−^ conjugate pair can subsequently oxidize ascorbate and participate in secondary radical propagation. Thus, Ar favors energetic bubble collapse but does not sustain oxygen-dependent radical pathways, whereas air provides both cavitation activity and O_2_-mediated secondary chemistry. This combination is consistent with the higher operational dosimetric response observed under air than under Ar.

Atomic oxygen may provide an additional connection between the dissolved gas atmosphere and the AscA response. At the high temperatures reached during bubble collapse, O atoms can be generated from water-derived species under all investigated atmospheres and, under air, additionally through thermal dissociation of O_2_. Simulations indicate that the amount of O formed depends strongly on the bubble temperature, vapor fraction, and gas composition [5]. Therefore, the higher AscA oxidation rate under air may include a contribution from atomic-oxygen chemistry in addition to the HO_2_^•^/O_2_^•−^ pathways discussed above. Nevertheless, because the O-atom yield and electronic-state distribution were not measured under the present reactor conditions, the observed gas order cannot be assigned quantitatively to atomic oxygen.

Argon enhances cavitation collapse because it is monatomic and does not dissipate collapse energy through rotational or vibrational molecular modes. This can increase bubble collapse temperature and promote water vapor dissociation [7], [28], [29]. However, in the present AscA system, air produced faster oxidation than Ar. This indicates that dissolved oxygen plays a decisive role in extending oxidative pathways beyond primary water sonolysis. Oxygen can react with hydrogen atoms to form hydroperoxyl radicals and can support secondary oxygen-derived radical chemistry:

(12) $${\text{H}}^{∙}+{\;\text{O}}_{2}\to {\text{HO}}_{2}^{∙}$$

H^•^ should not be considered solely as a precursor of oxygen-derived oxidants because it is also a cavitation-derived reducing species. Under aerated conditions, however, its competition with reductive pathways can be assessed kinetically. The reaction H^•^ + O_2_ → HO_2_^•^ is extremely fast in aqueous solution, with a bimolecular rate constant of approximately 2.1 × 10^10^ M^−1^ s^−1^ at room temperature [30]. The dissolved-O_2_ concentration in air-equilibrated water at 25 °C is of the order of 10^−4^ M and, although the presence of 0.2 M NaCl moderately decreases O_2_ solubility through salting out [18], an O_2_ concentration of approximately 2 × 10^−4^ M provides a conservative estimate for kinetic comparison. The corresponding pseudo-first-order H^•^ scavenging constant is therefore k′_H•+O2_ = k_H•+O2_[O_2_] ≈ 4 × 10^6^ s^−1^, equivalent to a characteristic H^•^ lifetime of approximately 2.5 × 10^−7^ s. This calculation is intended to establish the kinetic timescale of O_2_ interception; the local transient O_2_ concentration in the bubble–solution interfacial region was not measured. Thus, once H^•^ enters an oxygen-containing aqueous region, interception by O_2_ occurs on a submicrosecond timescale, forming HO_2_^•^ and subsequently contributing to the HO_2_^•^/O_2_^•−^ radical pool.

This kinetic competition strongly limits the fraction of H^•^ available for reductive reactions under aerated conditions, while simultaneously extending oxygen-mediated radical chemistry. Under Ar, by contrast, the O_2_-dependent pseudo-first-order sink is essentially absent, allowing a larger fraction of H^•^ to survive and participate in other interfacial or bulk-liquid reactions.

Because AscH^−^ is already the reduced member of the ascorbate redox couple, a possible reductive contribution of H^•^ would involve regeneration of AscH^−^ from its oxidized radical intermediate rather than further reduction of intact AscH^−^, i.e., Asc^•−^ + H^•^ → AscH^−^. Such regeneration would oppose the decrease in absorbance at 262 nm and therefore decrease the measured k_0,app_. However, the branching ratio between H^•^ scavenging by O_2_ and reaction with Asc^•−^ cannot be calculated quantitatively because neither the transient Asc^•−^ concentration nor the corresponding rate constant under the present sonochemical conditions was determined. Accordingly, the lower net AscA-depletion rate under Ar than under air is consistent with removal of the rapid H^•^ + O_2_ pathway and a consequently greater opportunity for H^•^-mediated reductive or termination reactions, but the magnitude of this contribution cannot be quantified from the present data.

Complementary experimental evidence for oxygen-dependent sonochemical pathways was reported by Nagata et al. [31], who observed gas-dependent ultrasonic degradation of hydroxybenzoic acids and showed that the contribution of oxygen under air became more important with increasing hydroxyl substitution.

In contrast, N_2_ produced the slowest oxidation. Nitrogen has a lower heat capacity ratio than Ar and can dissipate collapse energy through molecular degrees of freedom. Moreover, N_2_-saturated solution lacks oxygen-assisted radical propagation. These effects reduce the effective oxidative dose accessible to AscA.

Taken together, the gas-dependent trends indicate that the net AscA response is governed by the balance between cavitation-derived oxidative pathways and possible H^•^-mediated reductive pathways. Air favors O_2_-assisted oxidation and consumes reducing H^•^, whereas Ar may favor energetic collapse but also permit a greater H^•^ contribution. Because these pathways were not resolved independently, the observed gas order should be interpreted as a net operational response.

### 3.8 Competing oxidative and reductive pathways in the AscA dosimetric response

The proposed mechanism begins with water sonolysis during cavitation collapse:

(13) $${\text{H}}_{2}\text{O}\overset{\text{US}}{\to }{\text{HO}}^{∙}+{\;\text{H}}^{∙}$$

In addition to HO^•^ and H^•^, high-temperature reactions inside a collapsing bubble may generate atomic oxygen through further dissociation of water-derived species and, in aerated bubbles, through thermal dissociation of O_2_. Depending on the local temperature and chemical environment, the O atoms may be formed in the ground state, O(^3P), or an electronically excited state such as O(^1D). Numerical calculations predict appreciable O-atom formation during cavitation collapse, although the relative populations of O(^3P) and O(^1D) escaping into the liquid have not been established experimentally [5].

Hydroxyl radicals can recombine to form hydrogen peroxide:

(14) $$2{\text{HO}}^{∙}\to {\text{H}}_{2}{\text{O}}_{2}$$

or oxidize AscA/ascorbate:

(15) AscH^−^ + HO^•^ → Asc^•−^ + H_2_O

(16) AscH_2_ + HO^•^ → Asc^•−^ + H_2_O + H^+^

Because AscA has negligible volatility and a strong affinity for the aqueous phase, direct reaction between AscA and atomic oxygen within the bubble interior is unlikely. Any contribution from O atoms must therefore occur after their transfer to the bubble–solution interface or surrounding liquid. Excited O(^1D) is highly reactive toward water and is expected to remain confined mainly to the interfacial region, where its reaction with H_2_O produces additional HO^•^. In contrast, ground-state O(^3P) reacts much more slowly with closed-shell water molecules and may penetrate farther into the liquid phase. Its reported oxidation potential is comparable to that of HO^•^, making oxidation of the strongly reducing ascorbate species chemically plausible. Therefore, escaped O(^3P) may oxidize AscA directly or contribute indirectly through the formation of HO^•^, O_3_, or other oxygen-derived oxidants. However, because neither O atoms nor O-specific products were measured, their contribution cannot be separated quantitatively from that of HO^•^ in the present dosimetric response.

Thermal dissociation of water vapor also generates H^•^ atoms, which are reducing species. Their chemical fate, however, depends strongly on the dissolved-gas environment. As discussed in Section 3.7, H^•^ reacts with O_2_ in aqueous solution with k ≈ 2.1 × 10^10^ M^−1^ s^−1^ [30]. At the dissolved-O_2_ concentrations prevailing under aerated conditions, this corresponds to a pseudo-first-order removal rate of the order of 10^6^ s^−1^ and therefore to submicrosecond interception of H^•^ by O_2_. Consequently, H^•^-mediated reductive chemistry is kinetically strongly suppressed in oxygen-containing regions compared with oxygen-free conditions.

It is nevertheless important to distinguish possible reduction of AscA redox intermediates from reduction of intact AscA. AscH^−^ is already the reduced member of the ascorbate redox couple and is oxidized to Asc^•−^. A chemically plausible H^•^-mediated counterreaction is therefore radical–radical regeneration of ascorbate, Asc^•−^ + H^•^ → AscH^−^. H^•^ may also terminate HO^•^ through H^•^ + HO^•^ → H_2_O. Such pathways are expected to become comparatively more relevant under Ar, where the rapid H^•^ + O_2_ sink is absent. Because the transient concentrations of H^•^ and Asc^•−^ and the rate constant for their mutual reaction were not determined, their quantitative contribution cannot be established. The absorbance decay at 262 nm should therefore continue to be interpreted as a net operational response rather than as a direct measurement of the gross oxidative flux.

The rate constants for reactions of HO**^•^** with ascorbate species are close to 10^10^ M^−1^ s^−1^, making AscA an efficient radical scavenger [13], [16], [32]. The ascorbate radical can then undergo further oxidation or disproportionation to dehydroascorbic acid:

(17) 2Asc^•−^ + H^+^ → AscH^−^ + DHA

where DHA denotes dehydroascorbic acid. DHA may subsequently hydrate and hydrolyze to 2,3-diketogulonic acid and additional degradation products [13], [14], [15].

Hydrogen peroxide generated during ultrasonication may also oxidize AscA; however, its contribution is expected to be limited relative to HO^•^-initiated oxidation because H_2_O_2_ is substantially less reactive toward AscA than hydroxyl radicals [33]. Therefore, HO^•^ represents the best-established initiating oxidant for AscA under the present conditions because its reaction with AscA/ascorbate is close to the diffusion-controlled limit. Nevertheless, the measured depletion cannot be assigned exclusively to HO^•^. Possible contributions from escaped O(^3P), interfacial O(^1D)-derived HO^•^, H_2_O_2_, and secondary oxygen-derived radicals must also be considered, particularly under aerated conditions. AscA sono-dosimetry should consequently be interpreted as an operational measure of cavitation-derived oxidative activity rather than as a species-selective determination of HO^•^ alone.

Direct aerobic oxidation by molecular O_2_ should be distinguished from the oxygen-mediated sonochemical pathway. Ascorbate can undergo slow aerobic oxidation through ascorbyl-radical and O_2_^•−^/HO_2_^•^ intermediates, particularly in the presence of trace transition-metal species [34], [35]. Nevertheless, the absence of measurable AscA loss in the aerated non-sonicated control indicates that this background pathway was negligible under the present assay conditions. Consequently, the following reactions involving HO_2_^•^ and O_2_^•−^ represent mainly secondary species generated or sustained by sonochemical processes rather than appreciable oxidation by dissolved O_2_ before ultrasonication.

The positive and approximately linear depletion of AscA under all investigated conditions demonstrates that oxidative consumption dominated the overall dosimetric response. Nevertheless, the measured rate cannot be assigned exclusively to gross oxidation because H^•^-mediated reduction of Asc^•−^, and possibly other oxidized intermediates, may partially regenerate AscA. The reported k_0,app_, G_AscA_, and η_AscA,E_ values should therefore be interpreted as net operational indices of cavitation-derived oxidative activity.

Hydroperoxyl and superoxide radicals may also oxidize AscA:

(18) AscH^−^ + HO_2_^•^ → Asc^•−^ + H_2_O_2_

(19) AscH^−^ + O_2_^•−^ → Asc^•−^ + HO_2_^−^

However, their rate constants are several orders of magnitude lower than those for HO^•^ reactions [13], [16]. Thus, hydroxyl radicals are the primary initiating oxidants, while oxygen-derived secondary species contribute mainly under aerated conditions.

Overall, AscA sono-dosimetry reflects a coupled redox network rather than a single HO^•^ reaction. HO^•^ and possibly liquid-accessible atomic oxygen initiate AscA oxidation, while O_2_-derived HO_2_^•^/O_2_^•−^ and accumulated H_2_O_2_ may provide additional oxidative contributions. Conversely, H^•^ generated by water–vapor dissociation may reduce Asc^•−^ back to AscH^−^ and may terminate HO^•^. In aerated systems, H^•^ is preferentially converted into oxygen-derived species, shifting the balance toward oxidation, whereas oxygen-free Ar conditions may preserve a larger reducing H^•^ fraction. Since none of these transient species was quantified selectively, the UV–visible response is appropriately interpreted as the net cavitation-derived oxidative dose retained after competing oxidative, reductive, recombination, and radical-termination reactions.

### 3.9 Analytical performance of the AscA dosimetry method

The proposed method provides three complementary analytical response metrics: the direct absorbance decrease at 262 nm, the apparent zero-order oxidation rate constant k_0,app_, and the energy-normalized dosimetric response $\eta_{AscA,E}$. The high regression coefficients listed in Table 2 confirm the linearity of the C/C_0_ profiles, while triplicate experiments with RSDs below 2.5% demonstrate satisfactory repeatability.

A further analytical limitation concerns interpretation of the lower-UV spectral region. Several sonochemically generated inorganic and organic species may contribute to absorbance below approximately 230 nm, and the conventional UV spectra obtained here do not permit these overlapping contributions to be resolved. Accordingly, the lower-wavelength spectral changes were not used to identify individual oxidation products or to calculate any dosimetric parameter. Selective analyses would be required for product-specific characterization; for example, ion chromatography could be used to determine NO_2_^−^ and NO_3_^−^ if nitrogen oxyanion formation were of interest. This limitation does not affect the kinetic treatment employed in the present dosimetry method because residual AscA was determined from its characteristic analytical signal at 262 nm. The lower-UV spectra should therefore be regarded solely as qualitative evidence of chemical transformation rather than as product-specific identification.

The method is analytically simple because it relies only on UV–visible monitoring of the parent AscA signal. It does not require derivatization, fluorescence detection, chromatographic separation, or spin trapping. This makes it practical for routine comparison of sonochemical operating conditions.

The present approach should also be distinguished from the classical iodide competition method reported by Gutiérrez et al. [36]. In that study, the competition between HO^•^ recombination and HO^•^ scavenging by I^−^ or Br^−^ was used to infer a local HO^•^ concentration of approximately 4 × 10^−3^ M in the bubble–solution interfacial region. This approach provides valuable mechanistic information concerning the transient local radical concentration, but it requires concentration-dependent scavenging experiments, determination of multiple sonolysis products, and kinetic assumptions concerning the reaction zone. By comparison, AscA dosimetry uses the direct decrease of the parent-probe absorbance at 262 nm and can be readily expressed as time-, volume-, and calorimetric-energy-normalized indices. It therefore provides a simpler operational method for comparing the oxidative dose transferred from cavitation zones to a dissolved aqueous-phase solute. However, AscA depletion is not selective for HO^•^ and does not provide an absolute local HO^•^ concentration. The two approaches should consequently be considered complementary rather than interchangeable.

Compared with H_2_O_2_ accumulation, AscA dosimetry probes the oxidative dose accessible to a dissolved scavenger. This interpretation is consistent with comparative sonochemical dosimetry studies showing that ascorbic-acid-based dosimetry can provide a useful operational response in saline aqueous media [37]. H_2_O_2_ reflects radical recombination, whereas AscA depletion reflects the ability of cavitation-derived oxidants to react with a solute in the aqueous phase. The two methods are therefore complementary rather than equivalent.

The principal limitation of the AscA method is that the absorbance decrease at 262 nm is neither oxidant-specific nor a direct measurement of the gross oxidation rate. AscA may be consumed by HO^•^, oxygen-derived radicals, H_2_O_2_, and potentially liquid-accessible atomic oxygen. At the same time, cavitation-derived H^•^ may oppose this consumption by reducing Asc^•−^ back to AscH^−^, reducing other oxidized intermediates, or terminating HO^•^. Thus, k_0,app_ is a net apparent disappearance rate: r_AscA,net_ = r_oxidative consumption_ − r_reductive regeneration_. The method may consequently underestimate the gross oxidative flux when reductive regeneration is appreciable. It should be described as an operational dosimeter of the net cavitation-derived oxidative activity accessible to a dissolved solute, rather than as a selective assay of HO^•^ production. The method also depends on the composition of the stabilizing medium. Although 0.2 M NaCl effectively suppressed measurable background AscA loss in the present controls, its protection may depend on the concentration and identity of trace metals and on dissolved-O_2_ availability. Therefore, AscA stability should be reverified when the method is transferred to a different water source, salt concentration, reactor material, or complex aqueous matrix.

### 3.10 Implications for sonochemical reactor characterization

The proposed AscA dosimetry method provides an operational measure of the oxidative dose accessible to a dissolved solute. The combined use of k_0,app_, G_AscA_, and $\eta_{AscA,E}$ allows cavitation-derived oxidative activity to be compared on time-, energy-, and volume-normalized bases. This is useful because sonochemical activity depends not only on acoustic energy input but also on frequency-dependent bubble dynamics, gas atmosphere, reactor geometry, and radical transport from cavitation zones to the surrounding liquid. AscA dosimetry can therefore complement KI, Fricke, terephthalate, salicylic acid, and H_2_O_2_ dosimetry when comparing acoustic cavitation across reactors and operating conditions. Comparisons among reactors or gas atmospheres should also recognize that the AscA response may depend on changes in the oxidant-to-reductant balance. Two systems generating the same gross oxidant flux could produce different net AscA-depletion rates if their H^•^ yields or H^•^ escape efficiencies differ.

The present calibration applies to the reactor geometry, liquid volume, frequencies, and NaCl-stabilized AscA medium used in this work. Application to low-frequency probe systems, different sonoreactor geometries, or complex aqueous matrices should follow the same energy-normalized framework but requires independent calorimetric calibration and verification of AscA stability.

## 4 Conclusions

NaCl-stabilized AscA sono-oxidation was established as a simple UV–visible dosimetry method for evaluating cavitation-derived oxidative activity in ultrasonic systems. The addition of 0.2 M NaCl stabilized AscA against non-sonochemical oxidation, allowing the decrease in absorbance at 262 nm to be used as a reliable analytical signal during ultrasonication.

AscA depletion followed apparent zero-order kinetics under all investigated ultrasonic conditions, indicating that the rate was governed mainly by the quasi-steady generation and transfer of cavitation-derived oxidants rather than by the instantaneous AscA concentration. Increasing ultrasonic intensity accelerated AscA oxidation at 585, 860, and 1140 kHz.

Energy-density normalization showed that the oxidative response was not controlled only by cumulative acoustic energy input. The energy-specific AscA oxidation response decreased as frequency increased from 585 to 1140 kHz, showing that the energy-normalized AscA response was higher at 585 kHz than at 860 and 1140 kHz under the present reactor configuration and investigated operating conditions.

The pH results showed that the AscA dosimetric response was least sensitive to pH variation between pH 4.8 and 8, whereas pH 2 and pH 10 produced higher apparent oxidation rates due to changes in substrate speciation, interfacial distribution, and oxidation-product stability. Dissolved gas atmosphere strongly affected the response, with AscA oxidation following the order air > Ar ≫ N_2_. Atomic oxygen generated by high-temperature intra-bubble reactions may also contribute to the AscA response. Because AscA remains in the aqueous phase, such a contribution would arise from O atoms reaching the bubble interface or surrounding liquid rather than from direct intra-bubble oxidation. The relative contribution of atomic oxygen could not be isolated in the present study and remains an important subject for probe-specific mechanistic investigations. The stability of the aerated non-sonicated control demonstrated that this enhancement was not caused by appreciable background oxidation of AscA by dissolved molecular O_2_. Instead, the higher response under air is attributed to O_2_-mediated sonochemical radical pathways, including the formation and subsequent reactions of HO_2_^•^/O_2_^•−^.

AscA depletion provides a net rather than gross measure of cavitation-derived oxidative activity. Although oxidation dominated under all investigated conditions, H^•^ atoms generated through water–vapor dissociation can in principle counteract AscA loss through reductive regeneration of Asc^•−^ or radical termination. Under aerated conditions, however, the near-diffusion-controlled reaction of H^•^ with dissolved O_2_ provides a rapid pseudo-first-order sink on a submicrosecond timescale, strongly limiting the availability of H^•^ for these reductive pathways. Such reactions are therefore expected to become comparatively more relevant under Ar, where the O_2_-dependent H^•^ scavenging pathway is absent. Since the individual pathways were not quantified, the proposed method should be interpreted as an operational, energy-normalized measure of the net oxidative dose accessible to a dissolved solute.

Comparison with H_2_O_2_ generation confirmed that AscA sono-oxidation provides complementary information to radical recombination-based dosimetry. Because AscA may react with HO^•^, secondary oxygen-derived species, H_2_O_2_, and potentially liquid-accessible O(^3P), the method should be interpreted as an operational dosimeter of cavitation-derived oxidative capacity rather than as a selective measurement of HO^•^ alone. Nevertheless, AscA sono-oxidation provides a simple, reproducible, and energy-normalized platform for comparing sonochemical activity in acoustic cavitation systems.

## CRediT authorship contribution statement

**Oualid Hamdaoui:** Investigation, Conceptualization, Data curation, Methodology, Formal analysis, Resources, Validation, Project administration, Supervision, Funding acquisition, Visualization, Writing – original draft, Writing – review & editing. **Intissar Gasmi:** Investigation, Visualization, Validation. **Muthupandian Ashokkumar:** Validation, Supervision, Visualization. **Lahssen El Blidi:** Validation, Visualization, Resources. **Abdulaziz Alghyamah:** Validation, Visualization, Resources.

## Declaration of competing interest

The authors declare that they have no known competing financial interests or personal relationships that could have appeared to influence the work reported in this paper.
